# Development of Genomic Resources and Identification of Genetic Diversity and Genetic Structure of the Domestic Bactrian Camel in China by RAD Sequencing

**DOI:** 10.3389/fgene.2020.00797

**Published:** 2020-07-30

**Authors:** Chenmiao Liu, Huiling Chen, Zhanjun Ren, Xuejiao Yang, Chengdong Zhang

**Affiliations:** College of Animal Science and Technology, Northwest A&F University, Xianyang, China

**Keywords:** RAD sequencing, Bactrian camel, single nucleotide polymorphisms, genomic resource, genetic analysis

## Abstract

The domestic Bactrian camel is indispensable to agricultural production in the desertification area of China owning to its endurance to hunger and thirst, cold resistance, drought resistance, and good long-distance transportation. Therefore, it is necessary to investigate the genetic diversity, genetic structure, and genes with important roles in the evolution of this species. In this study, 1,568,087 SNPs were identified in 47 domestic Bactrian camels inhabiting four regions of China, namely Inner Mongolia, Gansu, Qinghai, and Xinjiang, by restriction site associated DNA sequencing (RAD-seq). The SNP data were used for nucleotide diversity analysis (π) and linkage disequilibrium (LD) attenuation analysis to elucidate the genetic diversity of the domestic Bactrian camel in the four regions studied. Results showed that Xinjiang camels had the highest nucleotide diversity and the fastest decay rate of the LD coefficient; therefore, Xinjiang camels had the highest genetic diversity. Structure analysis, principal component analysis (PCA), and phylogenetic tree construction by the neighbor-joining (NJ) method showed that Qinghai camels clustered separately, at a larger phylogenetic distance from camels in the other regions. Through analyses of selection signals, it was found that the number of selected genes shared by Inner Mongolia camels, Qinghai camels, Xinjiang camels, and Gansu camels was 7, 24, 25, and 113, respectively. The shared selected genes of the domestic Bactrian camel in the four regions were further analyzed, and three shared genes (GRIA3, XIAP, and THOC2) of the domestic Bactrian camel in China were identified. Gene Ontology (GO) classification and Kyoto Encyclopedia of Genes and Genomes (KEGG) enrichment analysis were performed on the shared selected genes of the domestic Bactrian camel in all four regions studied. Across all regions, genes involved in the cellular process were the most abundant subcategory under biological process. Cell and cell part represented the main proportion of genes under cellular component. Binding represented the main molecular function. In addition, the shared selected genes of the domestic Bactrian camel in the four regions of China were significantly enriched in the long-term depression pathway. The research should enable further study of the genetic resources of the domestic Bactrian camel, as well as the conservation of these resources.

## Introduction

The domestic Bactrian camel plays an important role in economic activities and trade, as well as in national defense, and has cultural significance; therefore, this animal represents one of the most valuable livestock resources in China (Chen et al., 2018). With the impact of modern civilization, such as the growth of the automobile transportation industry and the destruction of the natural environment, the number of domestic Bactrian camels has been greatly reduced, from 618,600 in 1981 to 380,000 in present-day China. Therefore, it is essential to study the genetic diversity of the domestic Bactrian camel and conserve its germplasm resources (Ming et al., 2017). To date, a variety of studies have been conducted on the origin, domestication, and genetic diversity of the domestic Bactrian camel worldwide. Molecular clock analysis based on the complete mitochondrial genome sequences shows that the wild camel was not the direct ancestor of the domestic Bactrian camel. The domestic Bactrian camel is considered to be monophyletic in terms of its evolutionary origin, and to originate from a single wild population (Ji et al., 2009). The earliest evidence on the domestication of Bactrian camels is mainly derived from the northeast of Ilang and the south of neighboring Turkmenistan, especially in the Kopet Daghmountain region. Bactrian camel skeletons were found in the second cultural layer of the Anau ruins on the northern edge of the mountain range, dating back to 3500–3000 BC (Meadow and Zeder, 1978). Studies of the genetic diversity of the domestic Bactrian camel through analysis of mitochondrial sequence variation found that there were no significant genetic differences between populations in China, Russia, and Mongolia, indicating that a strong gene flow occurred due to the extensive movement of the domestic Bactrian camel (Ming et al., 2017). However, the use of RAD-seq to identify whole-genome single nucleotide polymorphisms (SNPs) for genetic analysis of the domestic Bactrian camel in the four regions of China has not been reported to date.

In the past few decades, the use of SNPs for studying species genetic diversity and population structure has become widespread (Ren et al., 2019). The rapid development of high-throughput sequencing technology has led to the use of restriction site-associated DNA sequencing (RAD-seq) as a relatively cost-effective way to identify a large number of SNPs throughout the genome (Miller et al., 2007; Davey et al., 2011). So far, RAD-seq has been widely applied to population genetics studies of a variety of species (Zhang et al., 2016). For instance, Díaz-Arce et al. (2016) used RAD-seq data to infer the phylogeny of tuna based on whole-genome nuclear markers. Ren et al. (2019) used the RAD-seq method to identify genome-wide SNPs, and studied the genetic diversity and population structure of four Chinese rabbit breeds.

In this study, we discovered genome-wide SNPs in the domestic Bactrian camel using the RAD-seq method. We then investigated the genetic diversity, genetic structure, and genes with important roles in the evolution of this species in four regions of China (Inner Mongolia, Gansu, Qinghai, and Xinjiang). The study clarified the phylogenetic relationship of the domestic Bactrian camel in China and explored the genomic resources of this species. And the SNP resources generated in this study offer a valuable tool for future genetics and genomics research of the domestic Bactrian camel.

## Materials and Methods

### Sample Collection and DNA Extraction

Forty-seven venous blood samples were obtained from seven populations of domestic Bactrian agricultural camels from four regions of China (Inner Mongolia, Gansu, Qinghai, and Xinjiang) (Table 1) to ensure that each sample was derived from a different family, and there was no kinship between individuals. All methods and experimental protocols of this study were performed in accordance with guidelines and regulations of the animal ethics committee of Northwest A and F University (China) and the National Natural Science Foundation of China (31172178) Animal Care and Use Committee. DNA samples were extracted following a standard phenol-chloroform extraction procedure (Sambrook and Russell, 2002) and were diluted to 20 ng/μL.

**TABLE 1:** Information for domestic Bactrian camels in the four sampled regions of China.

Number	Group	Population	Sampling site	Sample size
1	Xinjiang camel	Nanjiang camel	Wensu county, Xinjiang	7
		Beijiang camel	Qinghe county, Xinjiang	7
		Dongjiang camel	Mulei county, Xinjiang	7
2	Gansu camel	Hexi camel	Yongchang county, Gansu	5
3	Qinghai camel	Qinghai camel	Mohe, Qinghai	7
4	Inner Mongolia camel	Alashan camel	Alashan Left Banner, Inner Mongolia	7
		Sunite camel	Sunite Right Banner, Inner Mongolia	7
Total				47

### Construction and Sequencing of RAD Libraries

RAD-seq libraries were constructed in accordance with the modified protocol (Baird et al., 2008). In short, EcoRI (New England Biolabs) was used to digest genomic DNA (0.1–1 μg; from a single sample or pooled samples), and P1 adaptors were connected at the cutting site. Then, the samples were pooled, randomly sheared, and size-selected in sequential steps. After the second adaptors (P2) were added, the sequencing libraries were constructed using DNA fragments of 300–700 bp in length. Finally, the constructed libraries were sequenced using the Illumina HiSeq3000 platform, and 100 bp paired-end reads were generated.

### Quality Control, Read Mapping, and SNP Calling

Quality trimming generated using fastp is an indispensable step to ensure high confidence of variant calling (Chen et al., 2018). Applying three strict filtering criteria, raw reads were processed to obtain high-quality clean reads: (i) removing reads with ≥10% unidentified nucleotides (N); (ii) removing reads with >50% bases having phred quality scores of ≤20; and (iii) removing reads aligned to the barcode adapter.

The Burrows-Wheeler Aligner (BWA) was used to align the clean reads of each sample with the reference genome^1^ with the settings “mem 4 -k 32 -M”, where -k is the minimum seed length, and -M being an option used to mark shorter split alignment hits as secondary alignments (Li and Durbin, 2009). GATK’s Unified Genotyper was used to conduct variant calling on all samples (DePristo et al., 2011). GATK’s Variant Filtration with proper standards was used to filter SNPs (-Window 4, -filter “QD < 2.0 | | FS > 60.0 | | MQ < 40.0”,-G_filter “GQ < 20”).

### Genetics Analyses

First, we used the VCFtools software suite to study the overall read depth and chromosome distribution of all SNPs (Danecek et al., 2011). The minimum read coverage for a SNP to be called is 3×, and all non-completely missing polymorphic loci (-max-missing 1e-06-non-ref-af 1e-06) were used for counting. The nucleotide diversity (π) of the domestic Bactrian camel in four regions of China was calculated using the PopGenome software package^2^ (Pfeifer et al., 2014). In addition, we used the 1,568,087 identified SNPs to estimate the linkage disequilibrium (LD) attenuation trend by calculating the LD coefficient (*r*^2^) between two points in a range of sequence (typically <5 Mb). The more rapid the decay of *r*^2^, the higher the genetic diversity of population is; *r*^2^ values in the range of 0,1 represent the correlation between two points: if *r*^2^ is 0, there is no correlation between the two loci, whereas if *r*^2^ is 1, the two loci are completely correlated. “LD attenuation distance” was used to evaluate the speed of LD attenuation (Guo, 2012).

### Analyses of Population Structure

The genetic structure was studied by phylogenetic tree construction, principal component analysis, and analysis of population structure. Following the identification of SNPs, 1,568,087 SNPs were used to calculate the phylogenetic distance between populations. The phylogenetic tree was constructed using a neighbor-joining (NJ) method with the software Treebest (version 1.9.2) to determine the evolutionary relationship between populations. Bootstrap values were generated from 1,000 replications. After removing sites with a missing rate of 50% or more, the remaining 865,774 loci were used for PCA, which was carried out using the GCTA software in R to further study the population genetic structure between regions (Yang et al., 2011). The STRUCTURE program^3^ (in order to ensure the independence of SNP marks, we used PLINK software to filter 865,774 SNPs according to LD intensity, the remaining 12,046 loci were used for structure analysis, -indep-pairwise 250 10 0.1, 250 kb window, the step size of 10 SNPs, *r*^2^ is <1) was used for analysis of population structure. We predefined the number of genetic clusters from *K* = 2 to *K* = 6 (BURNIN = 5,000 times, NUMREPS = 100,000), and repeated each *K*-value three times (Liu et al., 2019). Next, we used the POPHELPER software^4^ to calculate the value of Δ*K*; then, we used the CLUMPP software^5^ to combine the results of three repetitions (Schraiber and Akey, 2015). The fixation index (Fst) was calculated according to the statistical function of Fst in the PopGenome software package to study the genetic diversity between different regions (Pfeifer et al., 2014). Fst can also be used to infer the genetic distance between different regions. After removing the site with a missing rate of 50% or more, the remaining 865,774 loci were used for Fst analysis. PLINK 1.9 was used to calculate the inbreeding coefficient (Fis) of the domestic Bactrian camel in four regions^6^. After obtaining the Fis value for each sample, the average value in the region was determined.

### Analyses of Selection Signals

The top 5% region was selected based on the interception of two different parameters, namely nucleotide diversity (π) (Nei and Li, 1979) and population differentiation index (*F*_*ST*_) (Danecek et al., 2011). Using the 50 kb sliding window method with a step size of 25 kb, the −log10 transform of Nei’s π was used to select the lower end of diversity windows, and these parameters were quantified by internal PERL Scripts. All related graphs were drawn using R scripts (R Core Team, 2013). With Gansu camels as the control group and Inner Mongolia camels as the selection group, the genes of Inner Mongolia camels under selection pressure were identified. With Qinghai camels as the control group and Inner Mongolia camels as the selection group, the genes of Inner Mongolia camels under selection pressure were identified. With Xinjiang camels as the control group and Inner Mongolia camels as the selection group, the genes of Inner Mongolia camels under selection pressure were identified. Venn diagrams were then used to determine the common genes under selection pressure in Inner Mongolia camels; these genes played a crucial role in the evolution of this group. Using the same comparison method, the common genes under selection pressure were identified in Gansu camels, Qinghai camels, and Xinjiang camels.

### Sequence Annotation and Enrichment Analyses

In order to further systematically elucidate the complex biological functions of the genes, the common genes of the domestic Bactrian camel under selection pressure, in the four regions of China investigated in this study, were mapped against both GO and KEGG databases. GO enrichment analysis was performed with WEGO software, and gene numbers were calculated for every term (Ye et al., 2006). KEGG enrichment analysis using the KEGG database^7^ and KOBAS software were performed to determine the statistical enrichment in KEGG pathways of the common genes of the domestic Bactrian camel under selection pressure in the four regions (Mao et al., 2005). The calculated *p*-value was subject to false discovery rate (FDR) correction, applying FDR ≤ 0.05 as the threshold. Pathways meeting this condition were defined as significantly enriched pathways in the abovementioned genes.

## Results

### RAD-Tag Sequencing and Data Filtering

RAD sequencing produced a total of 131.13G of raw data for 47 normal sequenced individuals prior to quality filtering, with an average of 2.79G per sample, ranging from 1.36 to 4.57G. After quality filtering of the sequence data, 129.24G of clean data (1.34G to 4.30G for each sample, with an average of 2.75G) were retained, representing an average effective mapping rate of 98.57%. We mapped 1,568,087 regions, and the average spacing of RAD regions on the draft camel genome assembly was 1201.39 bp. The percentage of high-quality clean reads was above 97.73%, and the number of reads on the alignment was mostly above 97.39%. Of the clean reads retained, an average of 19.01 million reads were retained for each sample. In short, our sequencing data showed a high phred quality (Q20 > 94%, Q30 > 87%), and the GC content was stable, at between 40.71 and 45.13% (Supplementary Tables S1–S4).

### Genetics Analyses

**FIGURE 1:**
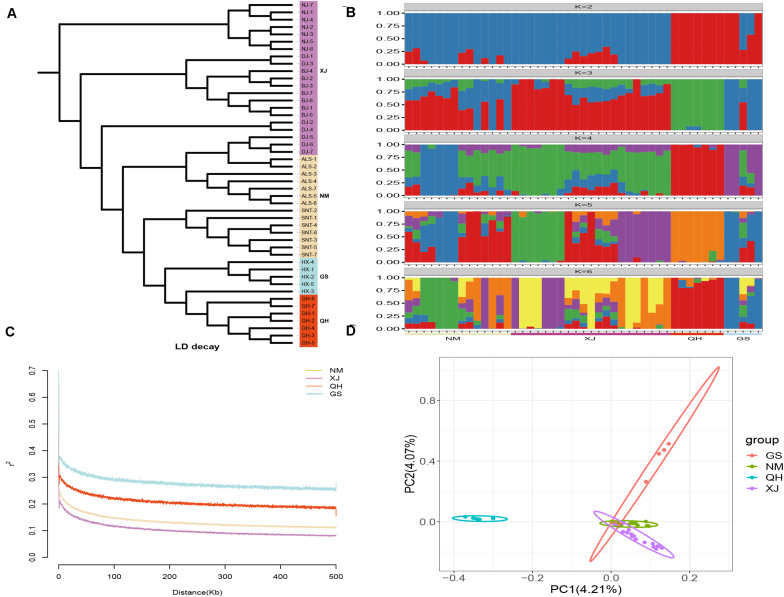
Structure analyses of the domestic Bactrian camel in four regions of China. **(A)** Phylogenetic tree construction with the neighbor-joining (NJ) method. **(B)** Groups structure clustering figure of the domestic Bactrian camel in four regions of China. **(C)** LD attenuation map of the domestic Bactrian camel in four regions of China. **(D)** Principal components analysis of the domestic Bactrian camel in four regions of China.

In this study, 1,568,087 SNPs were generated by RAD-seq. There were 548,830 loci (35%) in the transversion and 1,019,257 loci (65%) in the transition. The ratio of transition to transversion was close to 2:1. There were differences in the number of SNPs of the domestic Bactrian camel between the four regions, and the number of SNPs was in the following order: Xinjiang camels > Inner Mongolia camels > Qinghai camels > Gansu camels. The LD attenuation analysis of the domestic Bactrian camels from the four regions showed that the attenuation rate of the LD coefficient differed between the four regions, and that the attenuation rate was Xinjiang camels > Inner Mongolia camels > Qinghai camels > Gansu camels (Figure 1). Genome-wide nucleotide diversity was estimated from the SNP data. Because nucleotide diversity represents genetic diversity to an extent, it can be concluded from the data in Table 2 that the nucleotide diversity (π) of Xinjiang camels was the highest.

**TABLE 2:** Fst, π, and Fis values for domestic Bactrian camels in the four regions of China.

Number	Group	Average_Fst	π	Average_Fis
1	Xinjiang camel	0.07737	0.0001249	0.4473
2	Gansu camel	0.1201	0.0001091	0.4193
3	Qinghai camel	0.1185	0.0001088	0.3782
4	Inner Mongolia camel	0.08036	0.0001223	0.4434

### Population Structure Analyses

Genetic analysis of population structure using STRUCTURE software and PCA showed similar patterns. Cross-validation with *K* = 5 was the most suitable for the true differentiation history of the domestic Bactrian camel. At *K* = 5, the ancestral background of Qinghai camels was relatively pure, with a major genetic ancestor. Although the Inner Mongolia camels, Xinjiang camels, and Gansu camels had multiple genetic ancestors, a major genetic ancestor was evident. At *K* = 2, Qinghai camels were obviously separated from the camels in other regions, indicating that this group was phylogenetically distant from the camels in other regions. The PCA map showed that the Qinghai camels clustered together separately, and were phylogenetically distant from the camels in other regions. Inner Mongolia camels and Xinjiang camels gathered together, indicating that their genetic relationship was relatively close. The phylogenetic tree constructed by the NJ method showed that the domestic Bactrian camels of the four regions gathered together, and the branches of the tree were obvious. Xinjiang camels and Inner Mongolia camels gathered together (Figure 1). The Fst values were calculated to study the genetic distance between different regions. As shown in Table 2, it can be concluded that the average Fst between the Qinghai camels and the domestic Bactrian camels in other regions was 0.1185, second only to Gansu camels (0.1201), indicating a large genetic distance between Qinghai camels and other domestic Bactrian camels. It should be noted that the farther the kinship, the smaller the inbreeding coefficient. The average Fis between Qinghai camels and domestic Bactrian camels in other regions was the lowest, indicating a large genetic distance from camels in the other regions; these findings were consistent with the results of PCA and structure analysis.

### Analyses of Selection Signals

**FIGURE 2:**
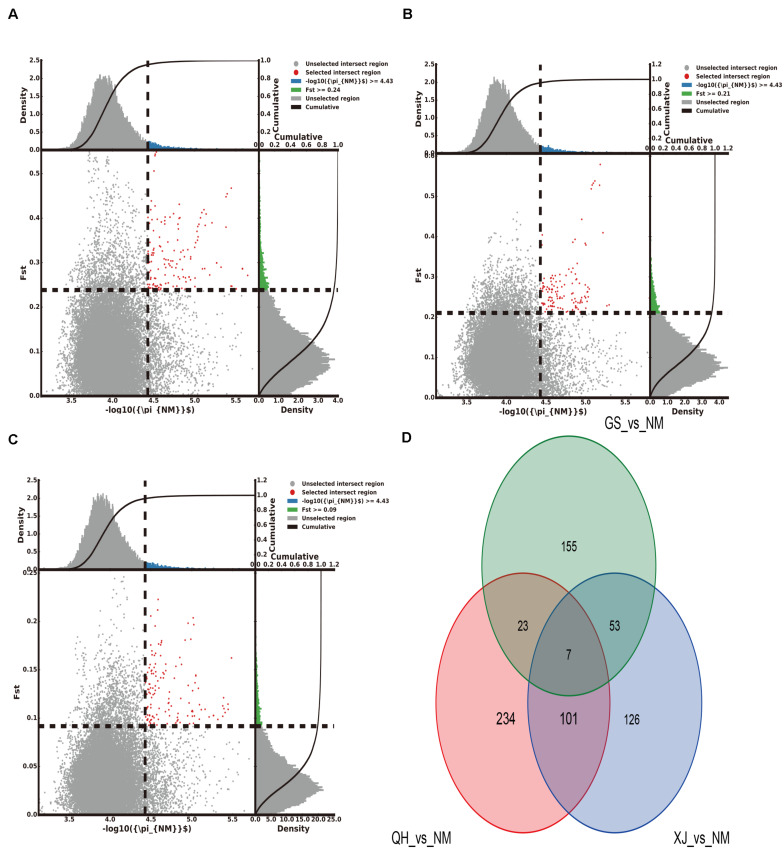
The number of selected genes shared by the Inner Mongolia group of camels. **(A)** Gansu camels were the control group and Inner Mongolia camels were the selection group; 238 selected genes were obtained. **(B)** Qinghai camels were the control group and Inner Mongolia camels were the selection group; 365 selected genes were obtained. **(C)** Xinjiang camels were the control group and Inner Mongolia camels were the selection group; 287 selected genes were obtained. **(D)** Seven selected genes were shared by Inner Mongolia camels.

The top 5% regions were selected by combining the π and the *F*_*ST*_. With Gansu camels as the control group and Inner Mongolia camels as the selection group, 238 selected genes were obtained. With Qinghai camels as the control group and Inner Mongolia camels as the selection group, 365 selected genes were obtained. With Xinjiang camels as the control group and Inner Mongolia camels as the selection group, 287 selected genes were obtained. Among them, 7 selected genes shared by Inner Mongolia camels were identified between all three comparisons (Figure 2 and Supplementary Tables S5–S7). Using the same comparison method, it was found that the number of selected genes shared by Qinghai camels, Xinjiang camels, and Gansu camels was 24, 25, and 113, respectively (Supplementary Figures S1–S3 and Supplementary Tables S8–S16). Venn diagrams were used to further analyze the shared selected genes of the domestic Bactrian camel between the four regions in China, and three shared genes (*GRIA3, XIAP, THOC2*) were identified in the domestic Bactrian camel across all four regions (Figure 7).

### Sequence Annotation and Enrichment Analyses

**FIGURE 3:**
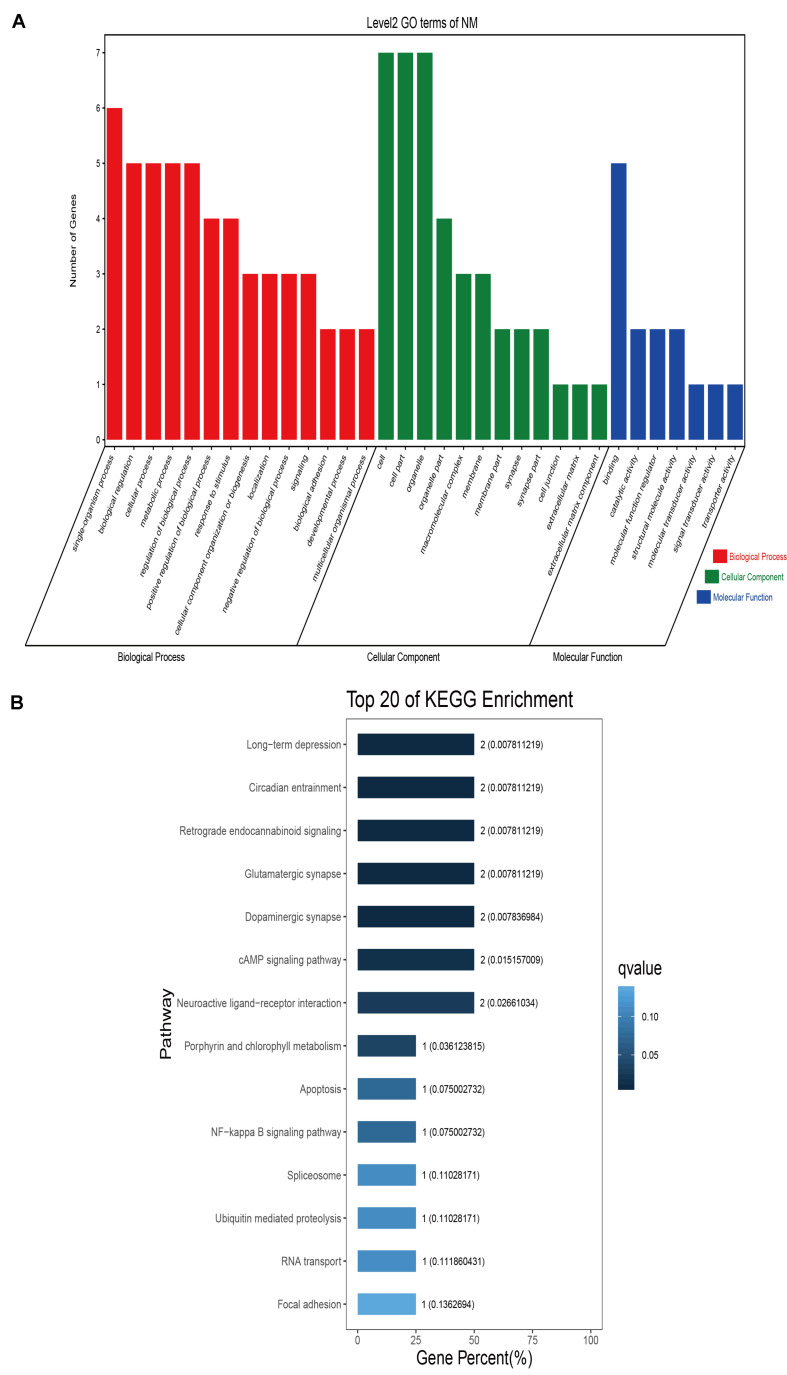
Go classification and KEGG enrichment of the shared selected genes of Inner Mongolia camels. **(A)** Go classification of the shared selected genes of Inner Mongolia camels. **(B)** KEGG enrichment of the shared selected genes of Inner Mongolia camels.

**FIGURE 4:**
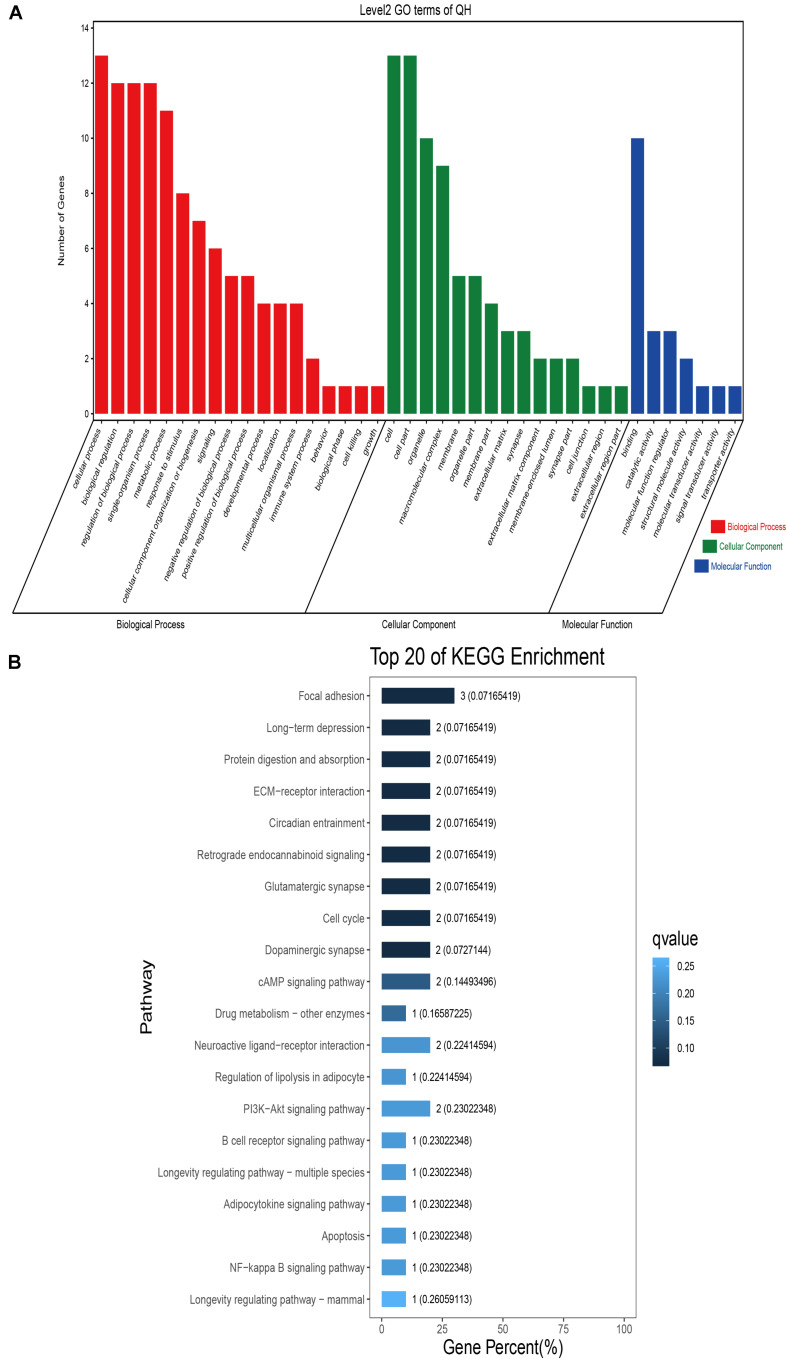
Go classification and KEGG enrichment of the shared selected genes of Qinghai camels. **(A)** Go classification of the shared selected genes of Qinghai camels. **(B)** KEGG enrichment of the shared selected genes of Qinghai camels.

**FIGURE 5:**
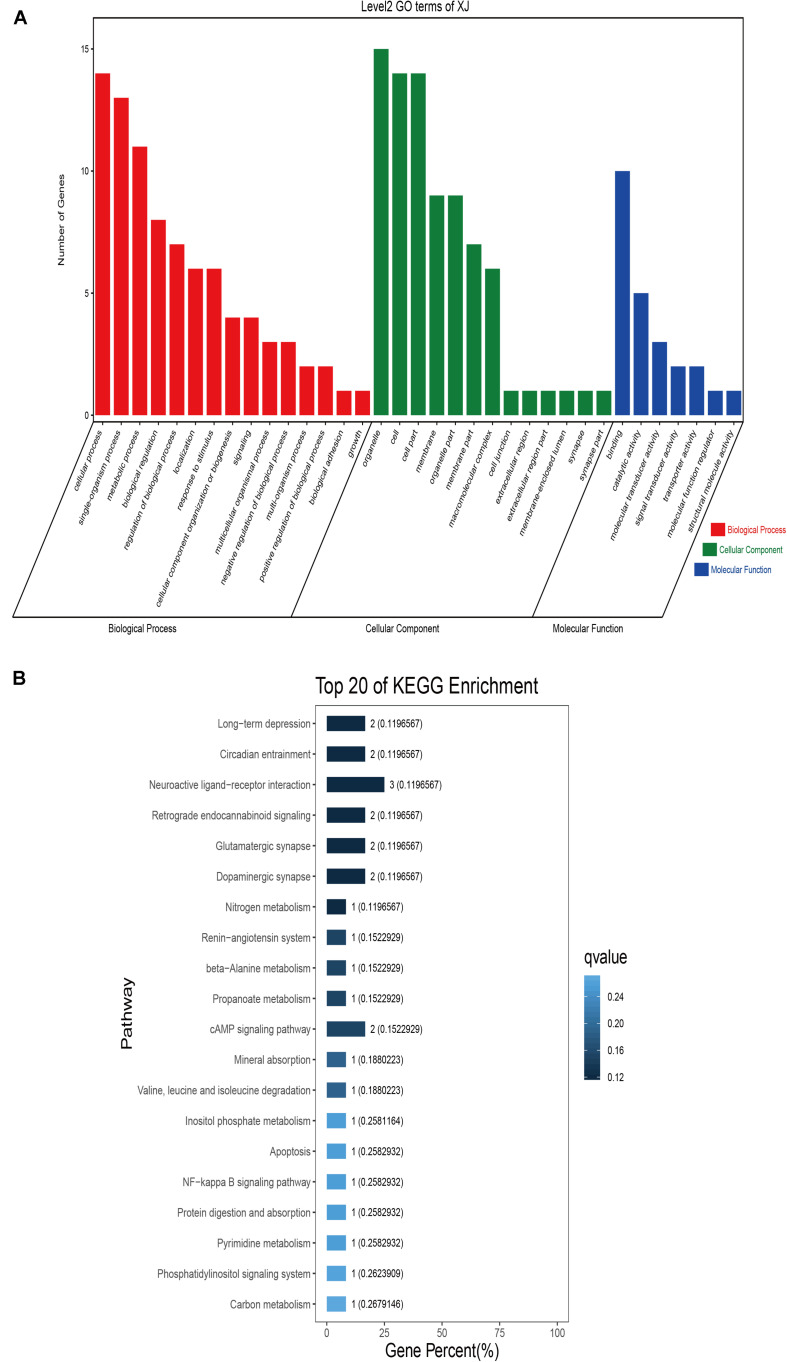
Go classification and KEGG enrichment of the shared selected genes of Xinjiang camels. **(A)** Go classification of the shared selected genes of Xinjiang camels. **(B)** KEGG enrichment of the shared selected genes of Xinjiang camels.

**FIGURE 6:**
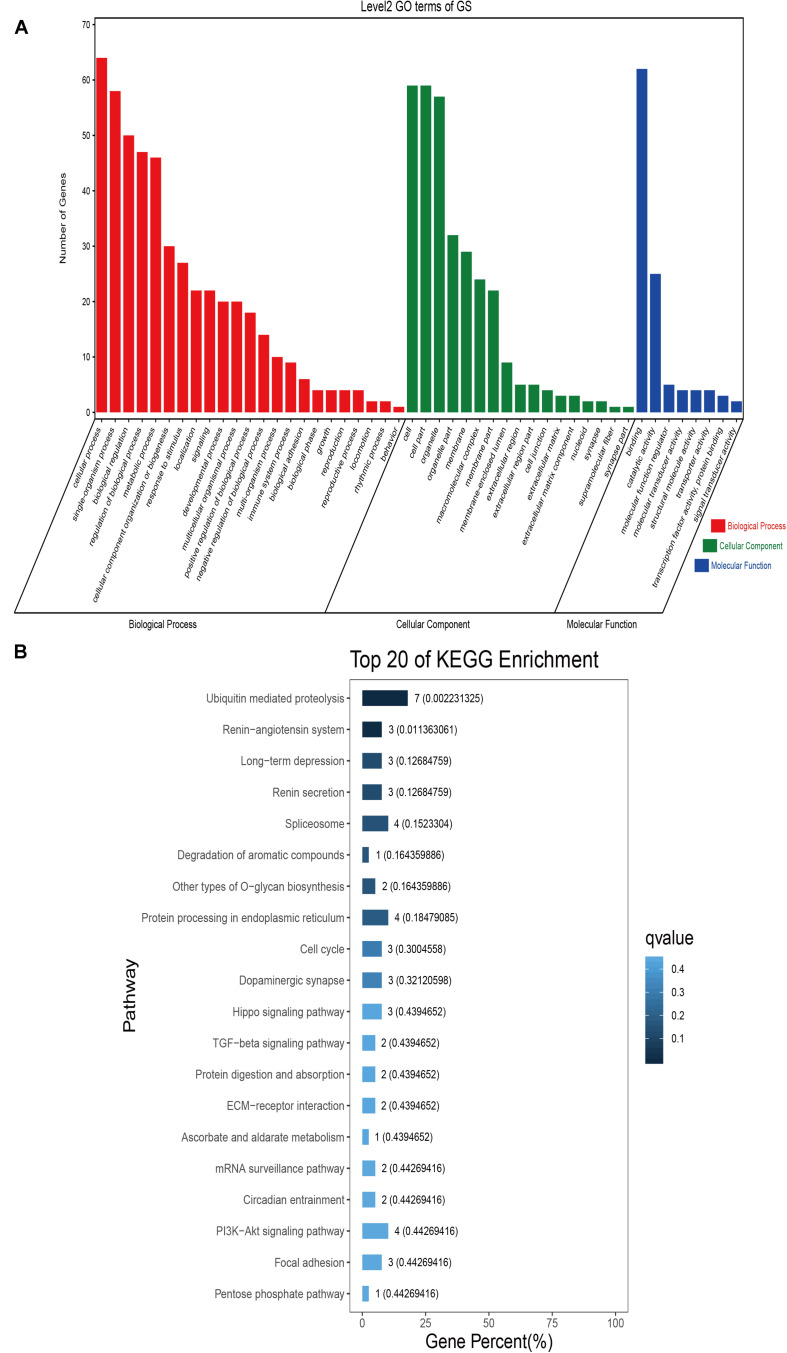
Go classification and KEGG enrichment of the shared selected genes of Gansu camels. **(A)** Go classification of the shared selected genes of Gansu camels. **(B)** KEGG enrichment of the shared selected genes of Gansu camels.

GO classification was carried out on the shared selected genes of the domestic Bactrian camel in the four regions. For Inner Mongolia camels, the genes involved in the single-organism process (GO: 0044699) were the most abundant under biological process. Cell (GO: 0005623), cell part (GO: 0044464), and organelle (GO: 0043226) represented the main proportion of the cellular component. Binding (GO: 0005488) accounted for a high proportion of the molecular functional category (Figure 3 and Supplementary Table S17). For Qinghai camels and Gansu camels, the most abundant subcategory under biological process was cellular process (GO: 0009987). Under the category of cellular component, the most abundantly expressed genes were cell (GO: 0005623) and cell part (GO: 0044464). Binding (GO: 0005488) represented the main molecular function (Figures 4, 6 and Supplementary Tables S18, S20). For Xinjiang camels, the genes involved in the cellular process (GO: 0009987) were the most abundant subcategory in biological process. Organelle (GO: 0043226) accounted for a high proportion under cellular component. Binding (GO: 0005488) represented the main molecular function (Figure 5 and Supplementary Table S19).

**FIGURE 7:**
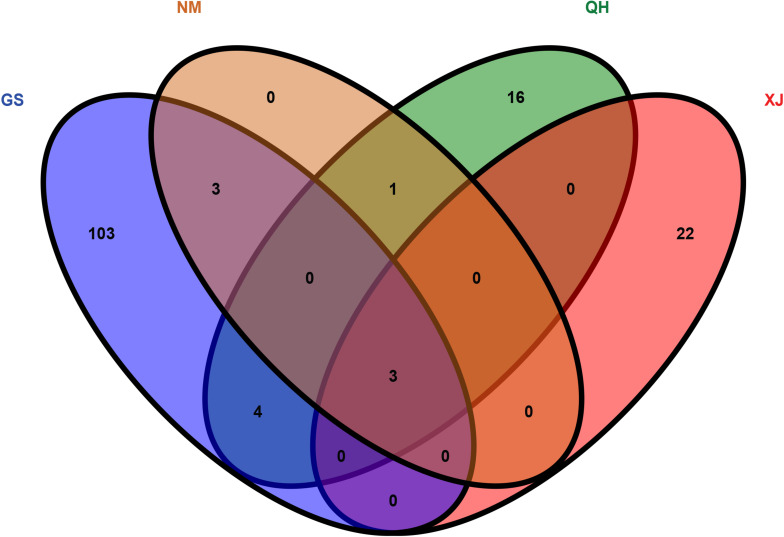
The shared selected genes of the domestic Bactrian camel in China.

KEGG enrichment analysis of the shared selected genes of the domestic Bactrian camel in the four regions was performed. Among the Inner Mongolia camels, the shared selected genes were significantly enriched in eight pathways, including long-term depression and circadian entrainment (Figure 3 and Supplementary Table S21). Among the Qinghai camels, the shared selected genes were mainly enriched in focal adhesion pathway (Figure 4 and Supplementary Table S22). Among Xinjiang camels, the shared selected genes were mainly enriched in neuroactive ligand-receptor interaction pathway (Figure 5 and Supplementary Table S23). Among the Gansu camels, the shared selected genes were significantly enriched in ubiquitin mediated proteolysis and renin-angiotensin system pathway (Figure 6 and Supplementary Table S24).

## Discussion

We reported the identification of 1,568,087 SNP loci in the domestic Bactrian camel in four regions of China, using RAD-seq. When read mapping, we used higher “*k*”-values, and the parameter settings are within the reasonable range of the software default (Cao et al., 2019; Shibuya and Comin, 2019; Liu et al., 2020). The filtered SNPs were subjected to LD attenuation analysis and nucleotide diversity analysis (π). The results indicated that Xinjiang camels had the largest number of SNPs, the fastest decay of the LD coefficient, the highest nucleotide diversity, and the highest genetic diversity: these features may be attributable to the preservation of the genetic diversity of this group’s ancestors (Abdulla et al., 2009). Population structural analysis, PCA, and Fis results suggested that Qinghai camels were phylogenetically distant from camels in other regions; this may be due to the geographical distribution of the Qinghai camels. The Mohe camel farm is located at an altitude of 3000 meters, which is far more elevated than the location of the domestic Bactrian camels in other regions, resulting in less genetic communication between Qinghai camels and domestic Bactrian camels in other regions. Phylogenetic trees constructed using the NJ method as well as PCA data revealed that the genetic relationship between Xinjiang camels and Inner Mongolia camels was relatively close. According to historical records, Inner Mongolia camels were extensively used to transport military supplies to Xinjiang in the Qing Dynasty, which may have promoted the genetic communication between Inner Mongolia camels and Xinjiang camels (Tong, 2018). The average Fst between Gansu camels and domestic Bactrian camels in other regions was the largest, and were phylogenetically distant from the camels in other regions. This may be related to the geographical location of Gansu camels. Yongchang County is located at the northern foot of the Qilian Mountain, close to the Badain Jaran desert, resulting in less genetic communication between Gansu camels and other domestic Bactrian camels. Analyses of selection signals showed that the number of selected genes shared by Inner Mongolia camels, Qinghai camels, Xinjiang camels and Gansu camels was 7, 24, 25, and 113, respectively. GO classification and KEGG enrichment analysis were performed on the shared selected genes. The results showed that the most abundant subcategory under biological process was cellular process (GO: 0009987). Cell (GO: 0005623), and cell part (GO: 0044464) accounted for a high proportion of the subcategories under cellular component. Binding (GO: 0005488) represented the main molecular function. The shared selected genes of domestic Bactrian camel populations in the four regions were all significantly enriched in the long-term depression pathway. This finding fills the gap in the genome study of the domestic bactrian camel in China (He et al., 2009; Ming et al., 2019).

The shared selected genes of the domestic Bactrian camel between the four regions in China were further analyzed to identify three shared genes (*GRIA3, XIAP, THOC2*) in the domestic Bactrian camel across all four regions. To our knowledge, this is the first study to identify genes of importance in the evolution of the domestic Bactrian camel in China. *GRIA3* (glutamate receptor 3) is the main excitatory neurotransmitter receptor in the mammalian brain, and is activated in many normal neurophysiologic processes. *GRIA3* regulates the activity of AMPA glutamate receptor and the NMDA receptor (Kato et al., 2010). Both AMPA receptors and NMDA receptors are classes of vital receptors in learning and memory (Brechet et al., 2017; Wang et al., 2018). According to the report of global human geography, the camel’s memory is second to none in all animals, and the ability of camels to remember routes gives these animals the capacity to navigate journeys accurately during sandstorms. *GRIA3* is therefore an interesting target for future research on the genome of the domestic bactrian camel in China. *XIAP* (E3 ubiquitin-protein ligase *XIAP*) is a multifunctional protein involved in the cellular response to DNA damage, and can also regulate inflammatory signal transduction and immunity (Deveraux et al., 1997; Damgaard and Gyrd-Hansen, 2011). The harsh environmental conditions of the habitat of the domestic Bactrian camel may promote apoptosis, DNA damage, and inflammatory reactions (Yuan et al., 2017). It can therefore be concluded that *XIAP* is an interesting target for future research on the genome of the domestic bactrian camel in China. *THOC2* (THO complex subunit 2) is a protein-coding gene that is involved in neuronal generation and neuronal development (Straesser et al., 2002). Mutations in this gene can cause hypotonia, gait disturbance, and tremors (Kumar et al., 2015). This gene is also an interesting target for future research on the genome of the domestic bactrian camel in China. A PPI network of the products of the three shared genes, constructed with STRING^8^, showed that there was interaction between the three encoded proteins (Supplementary Figure S4). Interestingly, the shared selected genes of the domestic Bactrian camel in the four regions were significantly enriched in the long-term depression (LTD) pathway. LTD has previously been assigned an assistant role in signal-to-noise adjustment or “forgetting”. However, LTD also contributes directly to the storage of hippocampal information (Collingridge et al., 2010). Furthermore, LTD plays a dominant role in the processing of precise spatial features. Increasing evidence supports the notion that LTD enables distinct and separate forms of information storage, which together promote the generation of a spatial cognitive map (Kemp and Manahan-Vaughan, 2007).

In brief, we identified the SNPs present in the domestic Bactrian camel genome on a whole-genome basis, and systematically studied the genetic diversity, genetic structure, and genes of importance in the evolution of the domestic Bactrian camel in four regions of China. The results should enable further study of the genetic resources of this mammal, as well as the conservation of these resources. In future studies, we aim to collaborate internationally to collect blood samples from camel populations inhabiting other regions along the Silk Road in order to further explore the available genome resources of this species.

## Data Availability Statement

The datasets generated for this study can be found in the NCBI SRA. Bioproject #PRJNA522647 and Biosamples #SAMN10948548–SAMN10948594.

## Ethics Statement

The animal study was reviewed and approved by National Natural Science Foundation of China (31172178). Written informed consent was obtained from the owners for the participation of their animals in this study.

## Author Contributions

ZR and CL designed the study and wrote the manuscript. HC collected the samples. CZ and XY contributed to the data analysis. All authors read and approved the manuscript.

## Conflict of Interest

The authors declare that the research was conducted in the absence of any commercial or financial relationships that could be construed as a potential conflict of interest.

## Footnotes

1 https://www.ncbi.nlm.nih.gov/genome/10741

2 https://cran.r-project.org/web/packages/PopGenome/vignettes/Whole_genome_analyses_using_VCF_files.pdf?tdsourcetag=s_pctim_aiomsg

3 http://web.stanford.edu/group/pritchardlab/structure.html

4 https://www.ncbi.nlm.nih.gov/pubmed/26850166

5 https://www.ncbi.nlm.nih.gov/pubmed/17485429

6 http://zzz.bwh.harvard.edu/plink/

7 http://www.genome.jp/kegg/

8 https://string-db.org/
