# Supplementary figures and images for: Development of Genomic Resources and Identification of Genetic Diversity and Genetic Structure of the Domestic Bactrian Camel in China by RAD Sequencing
Source: Front Genet. 2020 Jul 30;11:797. doi: 10.3389/fgene.2020.00797 (PMC7406665; doi:10.3389/fgene.2020.00797)

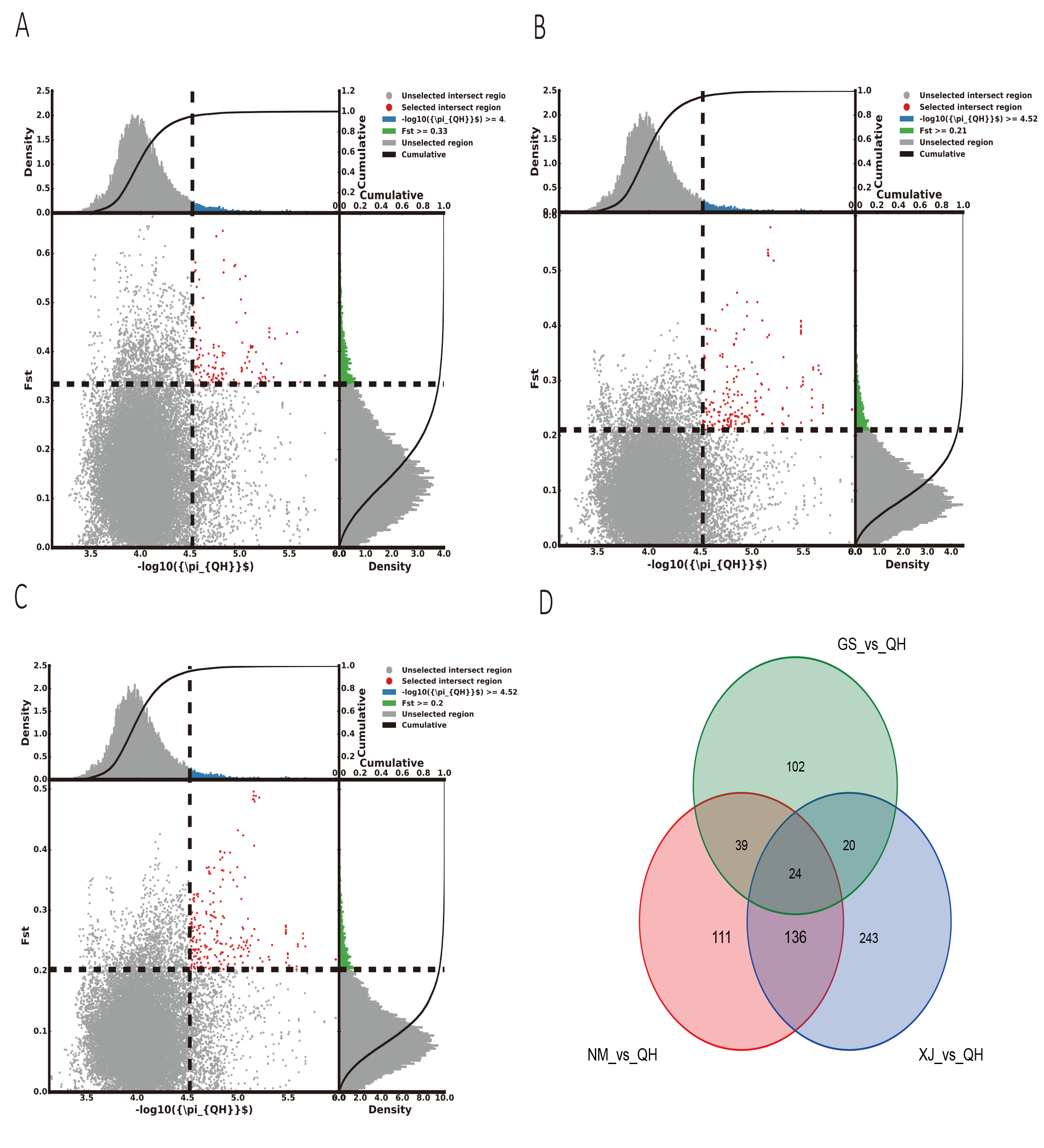

Supplement: Supplementary file 1 [file Image_1.TIF]

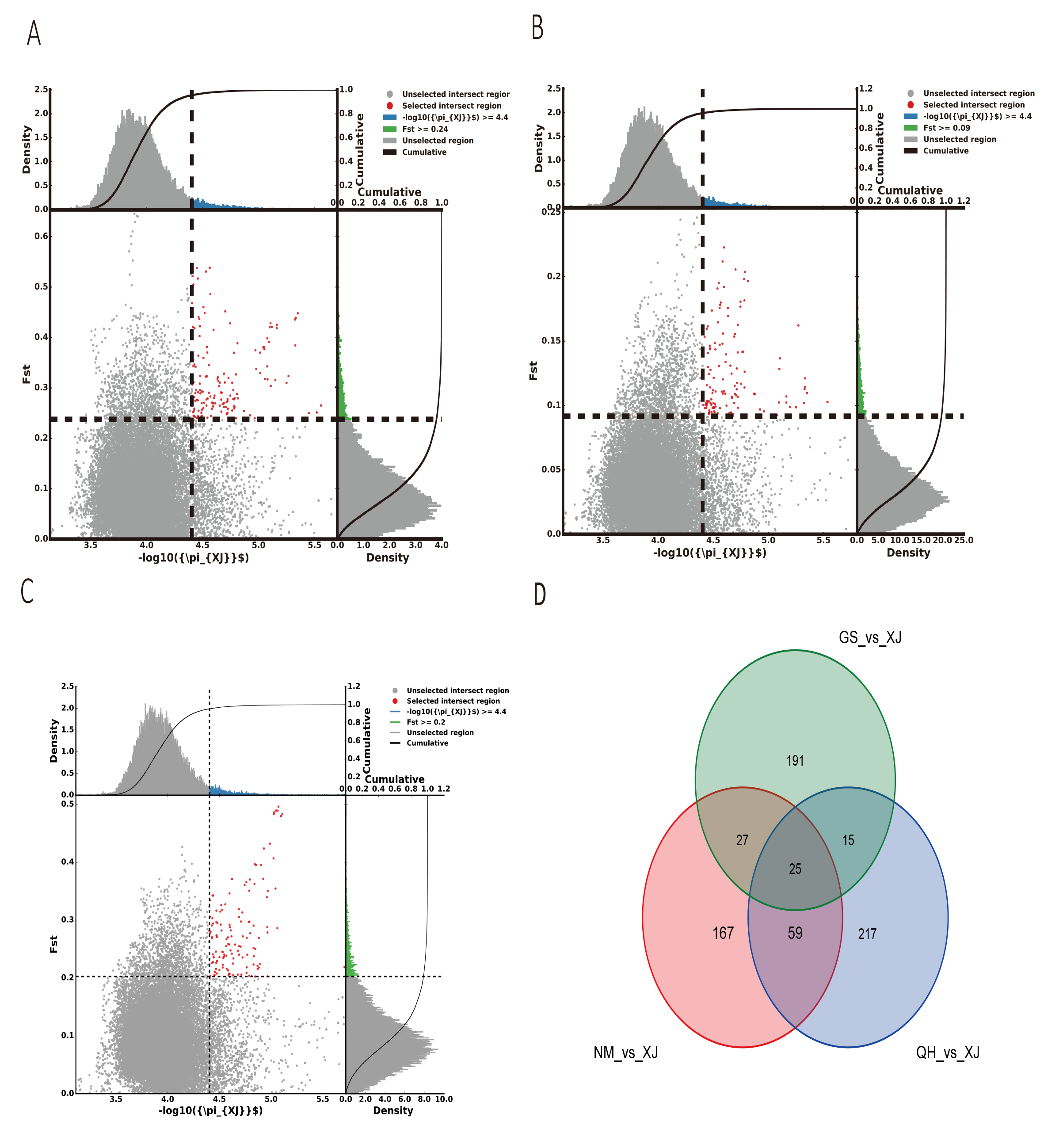

Supplement: Supplementary file 2 [file Image_2.TIF]

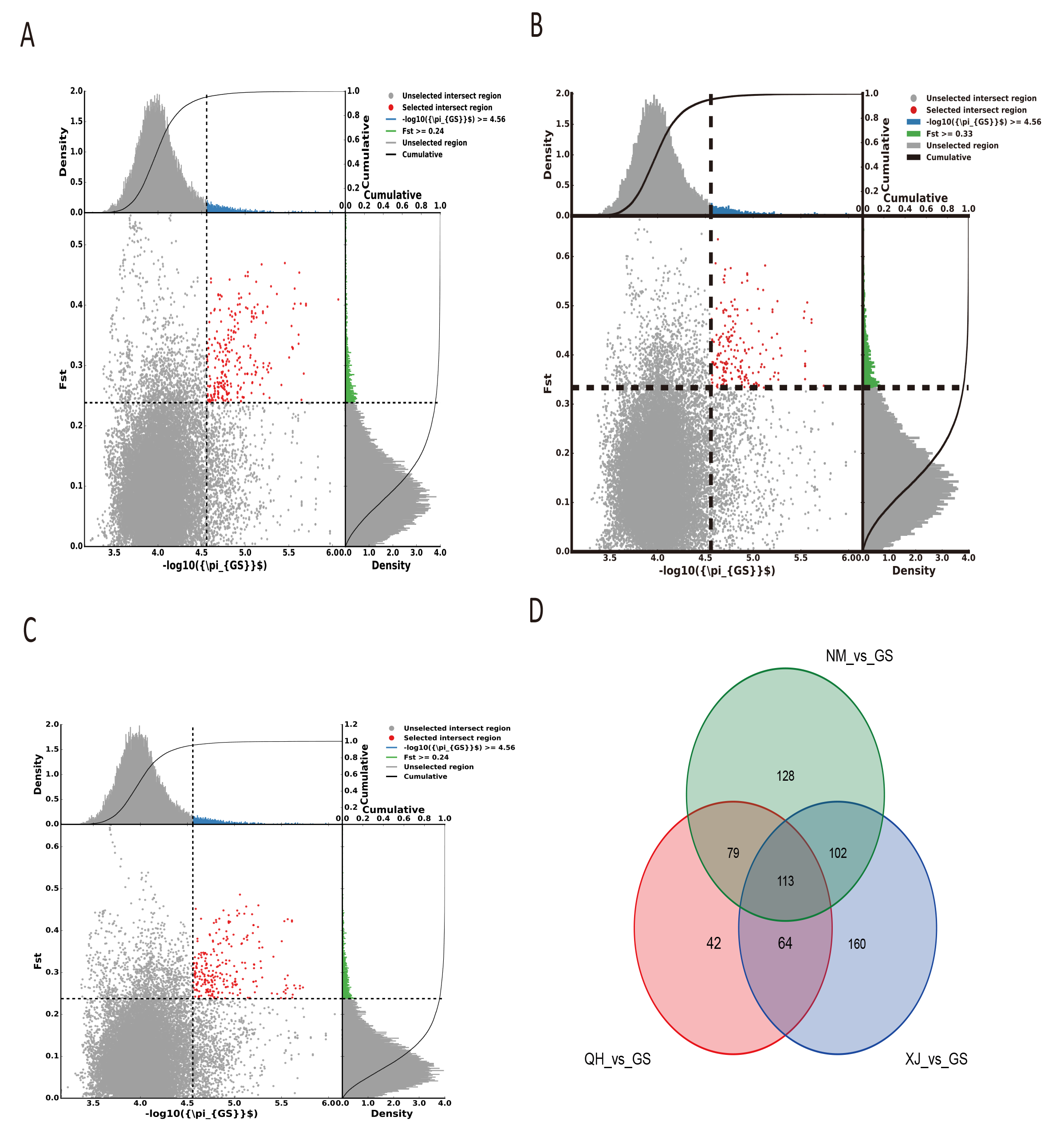

Supplement: Supplementary file 3 [file Image_3.TIF]

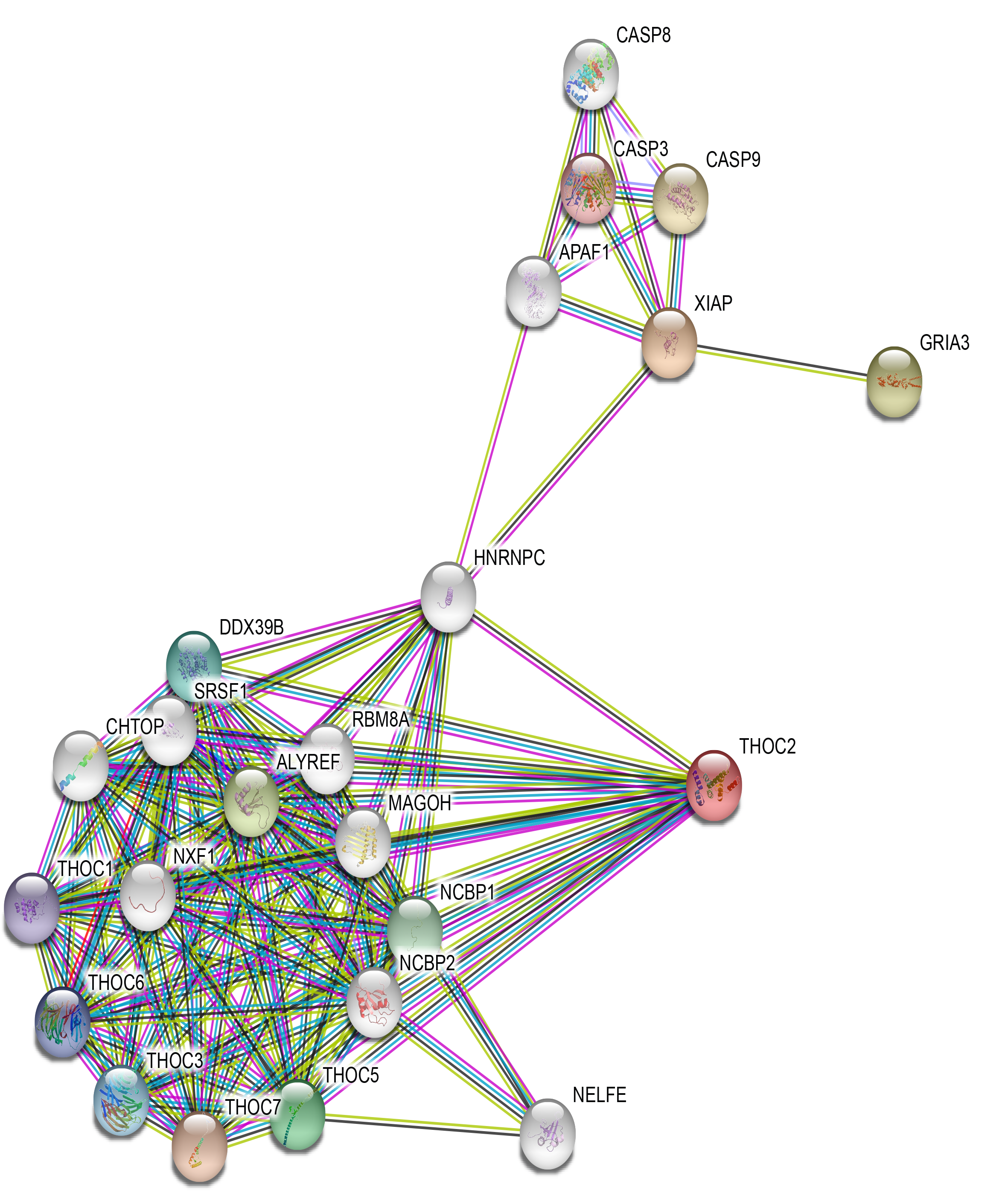

Supplement: Supplementary file 4 [file Image_4.TIF]
