# Supplementary material for: Development of Genomic Resources and Identification of Genetic Diversity and Genetic Structure of the Domestic Bactrian Camel in China by RAD Sequencing
Source: Front Genet. 2020 Jul 30;11:797. doi: 10.3389/fgene.2020.00797 (PMC7406665; doi:10.3389/fgene.2020.00797)

Supplementary Material

1 Supplementary Tables and Figures

1.1 Tables

Table S1. Sequencing results and quality filtering of reads.

Sample ID	Raw data (bp)	Clean data (bp)	Q20 (%)	Q30 (%)	GC (%)	Number of clean reads
**NJ-1**	1,923,714,618	1,888,444,546	94.54	87.72	41.86	12,849,068
**NJ-2**	2,256,475,284	2,217,140,920	94.66	87.97	41.86	15,085,772
**NJ-3**	1,669,436,744	1,638,753,132	94.54	87.77	41.79	11,075,836
**NJ-4**	2,338,084,760	2,296,297,744	94.67	88.00	41.56	15,519,716
**NJ-5**	2,783,092,640	2,732,847,981	94.65	87.98	41.45	18,469,790
**NJ-6**	3,388,941,710	3,330,190,644	94.76	88.19	40.96	22,582,460
**NJ-7**	1,853,499,541	1,818,839,943	94.49	87.65	41.96	12,418,044
**BJ-1**	2,527,717,739	2,475,830,840	94.36	87.45	40.79	16,903,804
**BJ-2**	2,436,141,468	2,393,113,567	94.63	87.91	41.47	16,339,060
**BJ-3**	2,279,005,980	2,235,896,582	94.49	87.65	41.65	15,318,494
**BJ-4**	2,442,658,840	2,397,555,661	94.61	87.94	40.77	16,425,076
**BJ-5**	2,034,006,592	1,997,661,570	94.62	87.92	41.34	13,685,584
**BJ-6**	1,921,363,504	1,887,000,434	94.48	87.62	42.05	12,927,946
**BJ-7**	1,361,748,132	1,337,196,198	94.52	87.69	41.76	9,161,260

**DJ-1**	1,904,026,880	1,871,441,712	94.68	88.03	41.35	12,822,720
**DJ-2**	3,121,809,012	3,089,768,436	97.14	93.23	41.58	21,023,556
**DJ-3**	1,818,269,754	1,800,523,106	97.17	93.28	41.54	12,251,464
**DJ-4**	2,869,579,944	2,842,384,026	97.17	93.29	42.68	19,341,436
**DJ-5**	4,567,389,695	4,304,910,992	95.98	89.53	40.71	30,513,168
**DJ-6**	3,507,693,832	3,473,079,253	97.15	93.22	42.85	23,474,096
**DJ-7**	3,261,057,752	3,228,334,579	97.12	93.17	42.83	21,820,692
**HX-1**	3,813,080,910	3,777,221,567	97.25	93.47	41.31	25,615,040
**HX-2**	3,520,284,832	3,485,153,272	97.15	93.25	41.83	23,795,788
**HX-3**	3,077,491,219	3,045,289,598	97.08	93.09	41.40	20,792,896
**HX-4**	3,454,365,985	3,421,343,897	97.20	93.35	41.68	23,360,230
**HX-5**	3,185,593,564	3,154,279,929	97.12	93.07	45.13	21,609,186
**QH-1**	3,566,197,068	3,523,728,005	96.83	92.58	41.83	23,977,968
**QH-2**	2,964,245,232	2,926,920,051	96.80	92.55	41.67	19,784,372
**QH-3**	3,921,322,752	3,872,581,201	96.82	92.55	41.72	26,174,636
**QH-4**	2,849,560,920	2,813,725,542	96.79	92.48	41.83	19,017,814
**QH-5**	3,041,945,600	3,005,488,473	96.82	92.52	42.71	20,384,078
**QH-6**	2,711,652,884	2,677,753,927	96.78	92.48	41.43	18,284,078
**QH-7**	3,031,866,138	2,992,531,538	96.70	92.30	41.49	20,432,936
**ALS-1**	3,073,842,536	3,043,333,590	97.16	93.27	42.20	20,850,150

**ALS-2**	3,511,102,900	3,477,671,146	97.22	93.40	41.37	23,825,024
**ALS-3**	3,323,513,632	3,292,014,703	97.18	93.31	41.68	22,553,956
**ALS-4**	3,178,993,196	3,149,054,922	97.23	93.35	42.77	21,573,390
**ALS-5**	3,017,539,972	2,989,868,222	97.26	93.46	41.81	20,483,570
**ALS-6**	2,888,820,480	2,851,489,760	96.76	92.43	41.52	19,402,950
**ALS-7**	1,544,276,748	1,525,212,252	96.76	92.39	42.43	10,378,418
**SNT-1**	3,328,148,617	3,288,134,940	96.83	92.56	41.52	22,451,738
**SNT-2**	2,598,194,976	2,564,381,067	96.76	92.43	41.34	17,568,912
**SNT-3**	2,409,331,420	2,379,650,806	96.82	92.56	41.48	16,303,460
**SNT-4**	2,140,263,056	2,114,152,181	96.73	92.39	42.08	14,486,496
**SNT-5**	3,498,269,500	3,457,098,448	96.83	92.55	41.44	23,684,982
**SNT-6**	2,988,436,624	2,952,583,045	96.84	92.58	41.40	20,228,716
**SNT-7**	2,224,795,596	2,199,168,964	96.88	92.63	42.31	15,067,196

Table S2. The average number of SNPs per Mb. (XLSX)

Table S3. Information about the RAD regions on the draft camel assembly. (XLSX)

Table S4. The coverage information of the estimated camel genome. (XLSX)

Table S5-S16. The results of the selection signals analyses. (XLSX)

Table S17-S20. Go classification of the shared selected genes of domestic Bactrian camels in four regions. (XLSX)

Table S21-S24. KEGG enrichment of the shared selected genes of domestic Bactrian camels in four regions. (XLSX)

1.2 Figures

Figure S1. The number of selected genes shared by Qinghai camels. (A) Gansu camels were the control group, Qinghai camels were the selection group, and 185 selected genes were obtained. (B) Inner Mongolia camels were the control group, Qinghai camels were the selection group, and 310 selected genes were obtained. (C) Xinjiang camels were the control group, Qinghai camels were the selection group, and 423 selected genes were obtained. (D) 24 selected genes shared in Qinghai camels.

Figure S2. The number of selected genes shared by Xinjiang camels. (A) Gansu camels were the control group, Xinjiang camels were the selection group, and 258 selected genes were obtained. (B) Inner Mongolia camels were the control group, Xinjiang camels were the selection group, and 278 selected genes were obtained. (C) Qinghai camels were the control group, Xinjiang camels were the selection group, and 316 selected genes were obtained. (D) 25 selected genes shared in Xinjiang camels.

Figure S3. The number of selected genes shared by Gansu camels. (A) Inner Mongolia camels were the control group, Gansu camels were the selection group, and 422 selected genes were obtained. (B) Qinghai camels were the control group, Gansu camels were the selection group, and 298 selected genes were obtained. (C) Xinjiang camels were the control group, Gansu camels were the selection group, and 439 selected genes were obtained. (D) 113 selected genes shared in Gansu camels.

Figure S4. The PPI network of three shared gene expression proteins.

2. The calculating formula of Nei's pi is:

$$\pi = \sum_{ij} x_i x_j \pi_{ij} = 2 * \sum_{i=2}^{n} \sum_{j=1}^{i-1} x_i x_j \pi_{ij}$$

Where xi and xj are the respective frequencies of the ith and jth sequences, πij is the number of nucleotide differences per nucleotide site between the ith and jth sequences, and n is the number of sequences in the sample.

3. The calculating formula of P-value is:

$$\mathrm{P} = 1 - \sum_{i=0}^{m-1} \frac{\binom{M}{i}\binom{N-M}{n-i}}{\binom{N}{n}}$$

Here N is the number of all genes with GO annotation; n is the number of genes in N; M is the number of all genes that are annotated to the certain GO terms; m is the number of genes in M.

$$P = 1 - \sum_{i=0}^{m-1} \frac{\binom{M}{i}\binom{N-M}{n-i}}{\binom{N}{n}}$$

Here N is the number of all genes that with KEGG annotation, n is the number of genes in N, M is the number of all genes annotated to specific pathways, and m is number of genes in M.

4. A mapping of the SNPs location across the chromosomes.

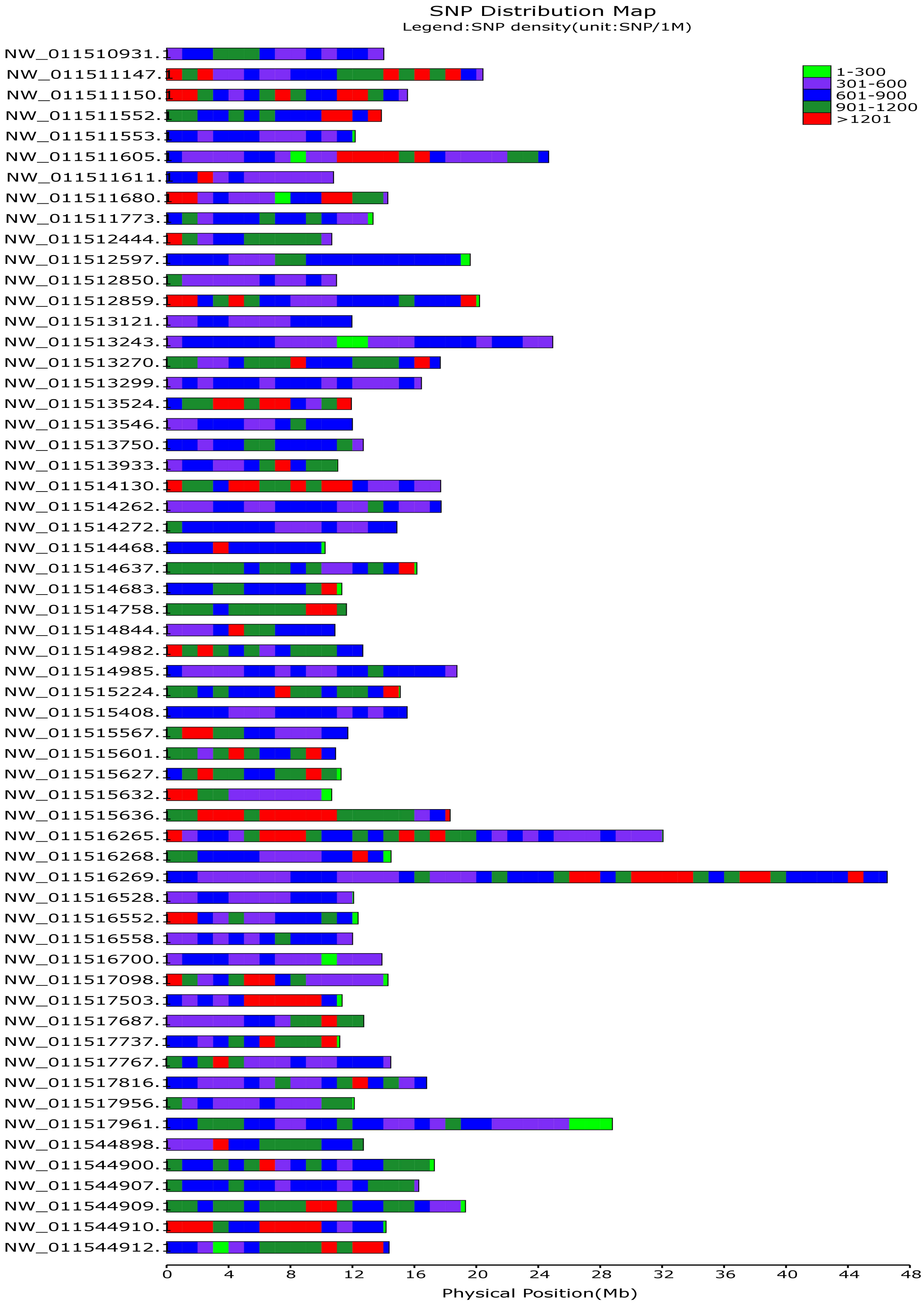

Supplement: Supplementary file 12 [file Data_Sheet_1.PDf]
